# Genome-Wide Analysis of Simple Sequence Repeats in Bitter Gourd (*Momordica charantia*)

**DOI:** 10.3389/fpls.2017.01103

**Published:** 2017-06-22

**Authors:** Junjie Cui, Jiaowen Cheng, Dingguo Nong, Jiazhu Peng, Yafei Hu, Weiming He, Qianjun Zhou, Narinder P. S. Dhillon, Kailin Hu

**Affiliations:** ^1^College of Horticulture, South China Agricultural UniversityGuangzhou, China; ^2^College of Agriculture, Guangxi UniversityNanning, China; ^3^BGI Genomics, BGI-ShenzhenShenzhen, China; ^4^General Station of the Administration of Seeds Guangdong ProvinceGuangzhou, China; ^5^AVRDC – The World Vegetable Center, East and Southeast Asia, Research and Training StationNakhon Pathom, Thailand

**Keywords:** bitter gourd, cucurbits, simple sequence repeats (SSRs), molecular markers, genetic diversity

## Abstract

Bitter gourd (*Momordica charantia*) is widely cultivated as a vegetable and medicinal herb in many Asian and African countries. After the sequencing of the cucumber (*Cucumis sativus*), watermelon (*Citrullus lanatus*), and melon (*Cucumis melo*) genomes, bitter gourd became the fourth cucurbit species whose whole genome was sequenced. However, a comprehensive analysis of simple sequence repeats (SSRs) in bitter gourd, including a comparison with the three aforementioned cucurbit species has not yet been published. Here, we identified a total of 188,091 and 167,160 SSR motifs in the genomes of the bitter gourd lines ‘Dali-11’ and ‘OHB3-1,’ respectively. Subsequently, the SSR content, motif lengths, and classified motif types were characterized for the bitter gourd genomes and compared among all the cucurbit genomes. Lastly, a large set of 138,727 unique *in silico* SSR primer pairs were designed for bitter gourd. Among these, 71 primers were selected, all of which successfully amplified SSRs from the two bitter gourd lines ‘Dali-11’ and ‘K44’. To further examine the utilization of unique SSR primers, 21 SSR markers were used to genotype a collection of 211 bitter gourd lines from all over the world. A model-based clustering method and phylogenetic analysis indicated a clear separation among the geographic groups. The genomic SSR markers developed in this study have considerable potential value in advancing bitter gourd research.

## Introduction

Over the past half century, the molecular markers utilized in research have shifted from enzyme-based to various DNA-based types. More than twenty kinds of DNA marker systems have been established (Agarwal et al., 2008). Of these marker types, simple sequence repeats (SSRs), which consist of short (typically 1–6 bp in length) tandemly repeated nucleotide motifs, have been recognized as the best choice for many types of research owing to features including their high variability and ubiquitous occurrence (Powell et al., 1996; Schlötterer, 2004). The development of SSR markers typically depends on the presence of SSR motifs and their flanking sequences in a species or taxon. Furthermore, the availability of a whole genome sequence of a particular species enables the genome-wide identification and development of SSR markers for that species (Cavagnaro et al., 2010; Song et al., 2015; Cheng et al., 2016).

Bitter gourd (*Momordica charantia*) belongs to the family Cucurbitaceae and is an important vegetable and medicinal herb in parts of Asia and Africa. The species has gained widespread attention as a result of its nutritional quality and incomparable hypoglycemic action, which has inspired the nickname ‘vegetable insulin’ for the crop (Khanna et al., 1981; Tan et al., 2008; Yang et al., 2015; Tan et al., 2016). However, since the whole genome sequence was previously unavailable, the development of large-scale SSR markers for bitter gourd has been limited. Only a small proportion of all potential bitter gourd SSR markers, including 26 developed using the fast isolation by AFLP of sequence-containing repeats (FIASCO) method (Wang et al., 2010; Guo et al., 2012), 171 developed from SSR-enriched fragments or libraries (Ji et al., 2012; Saxena et al., 2015), and 50 derived from transcriptomic data (Dhillon et al., 2016), have been reported publicly. This limitation has hindered the efficient genetic improvement of bitter gourd varieties via molecular marker assisted (MAS) approaches as well as many other types of research on the species.

We initiated the bitter gourd whole genome sequencing project in early 2014 and conducted *de novo* assembly of the approximately 294.0 Mb genome derived from the bitter gourd inbred line ‘Dali-11’ (unpublished). Utilizing the recently released ‘OHB3-1’ bitter gourd genome from Japan (Urasaki et al., 2017) as well, we report the characterization of genome-wide SSRs in bitter gourd, as well as SSRs from three other cucurbit genomes including cucumber (*C. sativus*), watermelon (*C. lanatus*), and melon (*C. melo*). The distribution frequency of SSR motifs among the four cucurbits were comprehensively determined and compared. Furthermore, a total of 138,727 unique SSR primer pairs were identified from the bitter gourd line ‘Dali-11’ genome, and 71 of them were validated by polymerase chain reaction (PCR) amplification. To verify their usefulness, 21 out of 71 unique SSR primer pairs were used to evaluate the genetic diversity of a collection of 212 *Momordica* samples with various origins. This work provides a valuable set of genome-wide SSR marker resources and will be useful for further molecular genetics applications in bitter gourd.

## Materials and Methods

### Plant Materials

A collection of 211 bitter gourd (*Momordica charantia*) samples and one *Momordica balsamina* sample (as an outgroup), originating from 16 countries as well as the World Vegetable Center (AVRDC) and seed companies, were used to validate the usefulness of SSR primer pairs that were identified in the present study (Supplementary Table S1). Apart from the 12 samples from AVRDC and 10 from seed companies, the other 189 bitter gourd samples can be divided into five distinct geographic origins, namely South Asia (47), Southeast Asia (62), Latin America (4), China (69), and Tanzania (7). Plants were grown in an open field at Zengcheng Teaching and Research Base, South China Agricultural University, Zengcheng (23°23′N, 113°64′E) in Guangdong Province, China.

### SSR Identification

The seven genomes from four cucurbit species are described in detail in Supplementary Table S2. The software package MISA^1^ was used to identify the SSRs from whole genome data. We constrained the length of SSR motifs to a range from 1 to 6 bp corresponding to mononucleotides (Mono-), dinucleotides (Di-), trinucleotides (Tri-), tetranucleotide (Tetra-), pentanucleotide (Penta-), and hexanucleotide (Hexa-), respectively. The detailed search criteria are described in our previously published study (Cheng et al., 2016) and were as follows: ten repeats for Mono-, six for Di-, and four each for Tri-, Tetra-, Penta-, and Hexa-, respectively.

### Development of Unique SSR Primer Pairs

In order to design primers flanking the SSR loci, two Perl scripts^2^ served as interface modules for the program-to-program data interchange between MISA and the primer modeling software Primer3 (Whitehead Institute, Cambridge, MA, United States). The general primer picking conditions were as follows: primer size, 18–27 bp with an optimum of 20 bp; primer melting temperature (*T*_m_), 57.0–63.0°C with an optimum of 60°C; product size, 100–500 bp with an optimum of 250 bp; and primer GC content, 40–60% with an optimum of 50%. Then all the designed primer pairs were aligned to the ‘Dali-11’ bitter gourd reference genome. Unique primer pairs were defined only if both the forward and reverse primers were uniquely aligned (with a 100% match rate) to the reference genome.

### Validation of SSR Markers by PCR Amplification

To validate the predicted motifs, we selected 27 primer pairs for SSR markers developed in two previous studies (Wang et al., 2010; Ji et al., 2012) and aligned these primers to the ‘Dali-11’ reference genome. To further validate the amplification of the unique set of SSR primers developed in this study, 50 primer pairs were randomly selected from 50 unassembled scaffolds (Supplementary Table S3). Each of the 71 primer pairs was used to amplify fragments from genomic DNA of two bitter gourd lines, ‘Dali-11’ and ‘K44.’ Genomic DNA was isolated from freshly collected leaf tissue samples using the cetyl trimethyl ammonium bromide (CTAB) method (Murray and Thompson, 1980). The PCR assay was conducted in a total reaction volume of 20 μL containing 20 ng of genomic DNA, 0.1 μM each forward and reverse primer, 0.1 mM dNTPs (Eastwin, Guangzhou, China), 0.5 U of Taq DNA polymerase (Eastwin, Guangzhou, China), 2.0 μL of 10× Taq buffer, and 2.0 mM MgCl_2_. PCR amplification was conducted under the following conditions: an initial denaturing step was performed at 94°C for 5 mins; followed by 25 cycles at 94°C for 30 s, 60°C for 30 s, and 72°C for 1 min; and finally followed by an extension step at 72°C for 5 min. The amplification products were resolved on a 6% polyacrylamide gel at 300 V for 2 h in 0.5× TBE buffer (pH = 8.3). Bands were visualized by silver staining.

### Genetic Diversity Analysis

After aligning 27 reported SSR markers to the ‘Dali-11’ reference genome, those markers that produced consistent motifs with our results were used to redesign markers for genotyping 212 *Momordica* samples. The model-based clustering program STRUCTURE2.3.4 (Pritchard et al., 2000), which involves posterior probability calculations of the data for a given *K*, Pr(*X*|*K*), was used to infer population groups. The statistic *K* was determined under the admixture model with correlated alleles, with a *K* that ranged from 2 to 10. Twenty independent runs of 10,000 Markov Chain Monte Carlo replicas and 100,000 generations of burn-in were used to estimate each *K* value. The optimal *K* value was determined by the log likelihood of the data [LnP(*D*)] in the STRUCTURE output and an *ad hoc* statistic Δ*K*, which was based on the rate of change in LnP(*D*) between successive *K* values (Evanno et al., 2005). The average number of alleles and polymorphic information content (PIC) were calculated by using the Powermarker V3.25 program (Liu and Muse, 2005). The neighbor-joining (NJ) algorithm appended in the MEGA6 (Tamura et al., 2013) was used to build the dendrogram based on the Nei1983’s genetic distances.

## Results

### SSR Motifs Content in Cucurbit Genomes

**FIGURE 1:**
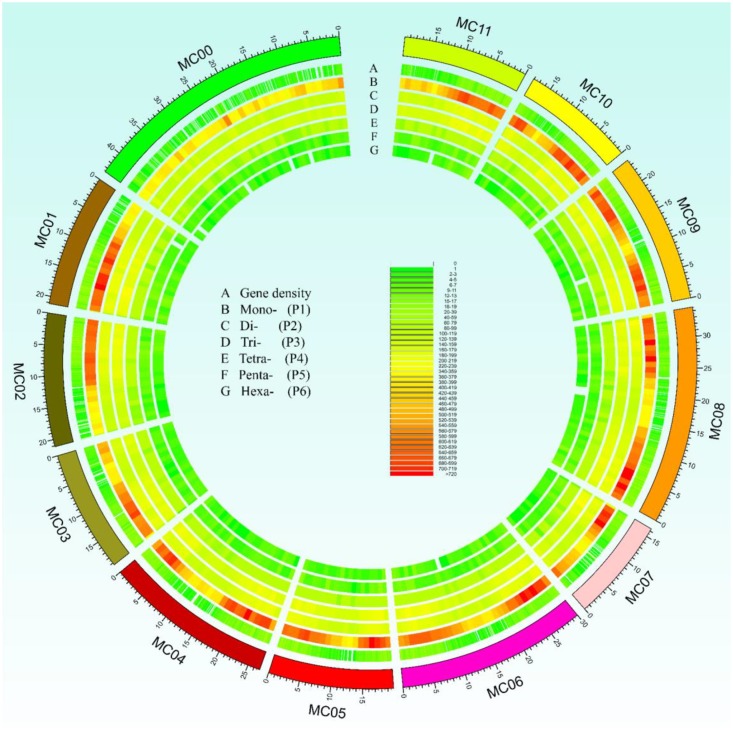
Overall view of simple sequence repeats (SSRs) motif distribution in the bitter gourd ‘Dali-11’ reference genome. A total of 188,091 SSR loci were identified in the ‘Dali-11’ reference genome. The unassembled scaffolds or contigs were assigned to MC00. Track A denotes the gene density; tracks B to G show the Mono-, Di-, Tri-, Tetra-, Penta-, and Hexa- repeats, respectively.

**FIGURE 2:**
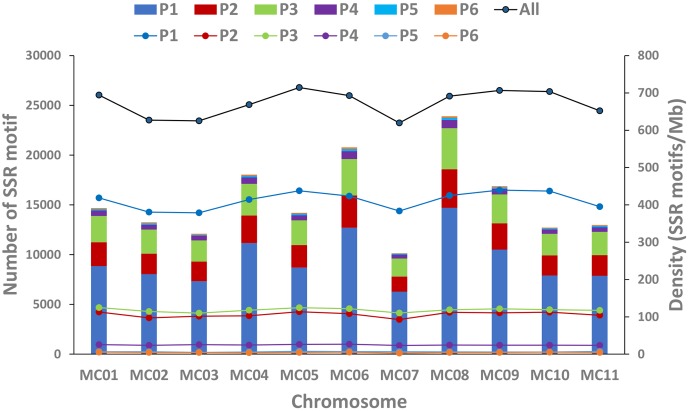
Number and density of SSR motifs across 11 chromosomes of the bitter gourd ‘Dali-11’ reference genome. Bars represent the numbers of SSR motif; Lines represent the density of SSR motifs. P1 to P6 indicate the Mono-, Di-, Tri-, Tetra-, Penta-, and Hexa- repeats; “All” indicates all SSR motifs.

In this study, a total of ∼2.15 Gb of sequence data from seven genomes of four cucurbit species, including two bitter gourd genomes, two cucumber genomes, two watermelon genomes, and one melon genome, was analyzed (Supplementary Table S2). According to the search criteria, we identified 129,947–331,062 SSR motifs among seven cucurbit genomes (Supplementary Tables S4–S8); 188,091 and 167,160 SSR motifs were identified within the ‘Dali-11’ and ‘OHB3-1’ genomes, respectively. SSR motifs in the ‘Dali-11’ genome are summarized in Supplementary Table S4 and **Figure 1**, and they occurred at higher frequencies within gene regions. The number of SSR motifs on each chromosome (MC01–MC11) ranged from 10,150 (MC07) to 23,915 (MC08; **Figure 2**). The number of SSR motifs identified in bitter gourd genome (mean = 177,625.50) was moderate in cucurbit species, which was higher than that found in cucumber genome (mean = 133,284.00), but lower than that of watermelon genome (mean = 313,711.00) and the melon genome (mean = 254,243.00; **Table 1**). Considering the different sizes of examined genomes, we calculated the density of SSR motifs for all cucurbit species. Bitter gourd genome, ‘Dali-11’ and ‘OHB3-1,’ had SSR densities of 639.73 and 585.27 SSRs/Mb, respectively, displaying comparable values to those of cucumber (658.72 and 667.08 SSRs/Mb for ‘9930’ and ‘PI183967,’ respectively) and melon (624.78 SSRs/Mb), but lower values than those of watermelon (834.24 and 818.07 SSRs/Mb for ‘97103’ and ‘WCG,’ respectively; **Table 1**).

**Table 1:** Frequency of various simple sequence repeats (SSR) motifs (1–6 bp in length) in seven cucurbit genomes.

SSR type	Genome						
	
	Dali-11 (*M. charantia*)	OHB3-1 (*M. charantia*)	9930 (*C. sativus*)	PI183967 (*C. sativus* var. *hardwickii*)	97103 (*C. lanatus*)	WCG (*C. lanatus*)	DHL92 (*C. melo*)
Mono-	114,789	98,499	69,085	71,731	187,528	208,537	136,601
Di-	29,648	29,048	25,117	27,470	37,951	43,200	44,597
Tri-	33,508	29,936	27,799	29,019	49,054	54,825	55,975
Tetra-	7,075	6,812	5,176	5,467	15,540	17,214	10,724
Penta-	1,776	1,671	1,665	1,768	4,205	4,941	4,550
Hexa-	1,295	1,194	1,105	1,166	2,082	2,345	1,796
Total number	188,091	167,160	129,947	136,621	296,360	331,062	254,243
Density^a^	639.73	585.27	658.72	667.08	834.24	818.07	624.78


*^a^Number of SSRs present in one million bases (SSRs/Mb).*

### Characterization of SSR Motifs in Cucurbit Genomes

Our search results revealed that Mono- repeats were dominant in cucurbit genomes, followed by Tri-, Di-, Tetra-, Penta-, and Hexa- repeats (**Table 1**). As the repeat number increased, the number of SSR motifs for each type was dramatically reduced (Supplementary Table S9). All SSR motifs could be classified into one of 424 kinds of motif types according to their occurrence (**Table 2** and Supplementary Table S10). Of these, 335 and 333 kinds were identified in ‘Dali-11’ and ‘OHB3-1’ bitter gourd genomes, respectively; both of which exhibited more kinds of motif types than the other cucurbit genomes. The theoretical number of motif types for Mono-, Di-, and Tri- repeats were 2, 4, and 10, respectively, and all 16 of these kinds of motif types appeared in the seven cucurbit genomes. Whereas, inter-specific differences in numbers of motif types occurred among Tetra-, Penta-, and Hexa- repeats (**Table 2**). Notably, the classified motif types were not completely uniform between the two genomes even belonging to the same species. For example, the two motif types AGCT/AGCT and CCCCCG/CGGGGG were identified in ‘Dali-11,’ but not detected in ‘OHB3-1.’

**Table 2:** Number of SSR motif types classified based on motif length.

Genome	Mono-	Di-	Tri-	Tetra-	Penta-	Hexa-	Total
Dali-11	2	4	10	33	77	209	335
OHB3-1	2	4	10	32	77	208	333
9930	2	4	10	32	67	193	308
PI183967	2	4	10	33	73	185	307
97103	2	4	10	31	71	194	312
WCG	2	4	10	32	71	192	311
DHL92	2	4	10	33	75	196	320
Total	2	4	10	33	90	285	424


**FIGURE 3:**
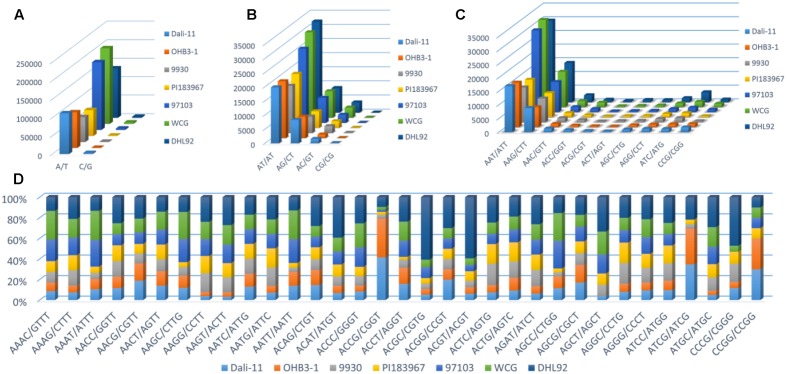
The number or percentage of classified SSR motifs in seven cucurbit genomes. **(A)** Mono-; **(B)** Di-; **(C)** Tri-; and **(D)** Tetra- repeats.

Furthermore, the frequency of classified SSR motifs in the four cucurbit species were variable (**Figure 3** and Supplementary Table S10). Briefly, with regard to the Mono-, Di-, Tri-, and Tetra- repeats, A/T, AT/AT, AAT/ATT, and AAAT/ATTT were overrepresented, respectively, which totally accounted for 63.86–76.30% of all motifs identified in the seven cucurbit genomes. The AAAAT/ATTTT motif was found to be the most frequent motif of Penta- repeats in bitter gourd and watermelon genomes, whereas AAAAG/CTTTT was the most frequent one in cucumber and melon genomes. The AAAAAG/CTTTTT and AAAAAT/ATTTTT motifs were most representative among Hexa- repeats, and their numbers were roughly uniform across bitter gourd and watermelon genomes, whereas the former motif was several times more numerous than the later in cucumber and melon genomes.

### Development and Validation of Unique SSR Primer Pairs

To advance bitter gourd research efforts, we retrieved the flanking sequences of all genomic SSR motifs and used them as targets for primer design. Based on the primer picking conditions, a total of 159,838 SSR primer pairs were successfully designed. To obtain unique primer pairs, we aligned them back to the ‘Dali-11’ reference genome and ultimately identified a set of 138,727 (86.79%) unique primer pairs (Supplementary Table S11).

We selected 27 pairs of primers for SSR markers from published reports and verified their motifs in the present study (**Table 3**). Overall, 23 out of 27 (85.19%) of these motifs were consistent, but markers N9 and N12 mapped to the same position within S18 and S15, respectively. Hence, we designed 21 unique primer pairs (**Table 3**). In combination with 50 additional randomly selected primers, each of the examined 71 primers produced clear bands in at least one of the two bitter gourd lines ‘Dali-11’ and ‘K44’ DNA samples, showing a 100% amplification rate (**Supplementary Figures S1**, **S2**).

**Table 3:** Validation of 27 SSR motifs and polymorphisms for 21 primer pairs in 211 bitter gourd samples.

Markers	SSR motifs	Markers from published reports	GenBank	Motifs from published reports	Primer sequences (5′–3′)	Allele No	PIC
–	–	JY001^∗^	JQ358823	(CT)5(CTT)17	–	–	–
–	–	JY002^∗^	JQ358824	(CTT)9	–	–	–
MC08_106502	(**AAG**)26	JY003	JQ358825	(**CTT**)22	F:TGCCAAGCAAATACCACAAA R:TGAAGAAGCAACAGCAGCAG	14	0.84
MC04_54893	(**GAA**)14	JY004	JQ358826	(**CTT**)8	F:GAAAGCGAGAACGAACGAAC R:TGCCATCGGTACTCATCAAA	9	0.74
MC05_69551	(**AG**)6	JY005	JQ358827	(**AG**)6(CTT)7	F:GGACGATCGACGTACGTTTT R:TAGCAAACGGCTCAAGCTCT	2	0.37
MC10_152791	(**TTC**)16	JY006	JQ358828	(**CTT**)16(CT)6	F:TTGCAGCTTCCTTTCTGGTT R:AGGAAACACATCTTGGGCTG	6	0.67
–	–	JY007∗	JQ358829	(CTT)11	–	–	–
MC10_147313	(**CT**)21	JY008	JQ358830	(**CT**)22	F:CGGCATGAAGAATGGCTAAT R:GGGGTTTTCCCCTAATTGAA	5	0.62
MC10_143173	(**GA**)26	JY009	JQ358831	(**CT**)23	F:TGTTGCCTTTGACTGCAAAT R:AGGCAAGAGGAATGGAGGTT	6	0.62
–	–	JY010∗	JQ358833	(CTT)13	–	–	–
MC07_95456	(**CT**)16	JY011	JQ358833	(**CT**)15	F:TTCTTGAGAGACGGTTGGCT R:GATACAAAGAAACGGTGGCG	7	0.64
MC04_46305	(**GA**)11(**A**)10	N1	GU166217	(**GA**)11(**A**)9	F:TCCGAGTTCAAACCAGTTCC R:ATCTGGTTCCTCGGGAGATT	3	0.54
MC03_37553	(**AG**)13	N5	GU166218	(**CT**)13	F:TCAATAATGCATTCTCCCCC R:AGGGGATTTCCACCAAAAGA	3	0.51
MC09_141866	(**TC**)14	N6	GQ338437	(**GA**)14	F:TGGCACACTCTGCATGAAAT R:TGTCAGAAGTTGGAACGACG	4	0.51
MC01_5661	(**AG**)15	N9	GU166219	(**TC**)17	F:GGGGTGGCTGGAATATATGA R:ATCCATCCCCACAAGTTGAA	5	0.57
MC08_111602	(**CT**)11tctt(**TC**)6	N12	GU166220	(**AG**)9(**GA**)11	F:GTGTTTTCGTGAGGGAGGAA R:TGGGTAGTGGAATGATGGGT	4	0.51
MC01_6583	(**GA**)13	N24	GU166221	(**GA**)13	F:GACGAGTTTAAAAGACTTTCGG R:TGACCAAGCAACCATGTGAT	3	0.49
MC04_42551	(**TC**)6ttcctt(**TC**)11	S9	GQ338438	(**TC**)6(**TC**)11	F:AGAAAGAGGGGGAAAGACCA R:AAGGCATCGTATGGGAAGTG	3	0.42
MC01_4761	(**GA**)13	S12	GQ338439	(**TC**)14	F:TAACGAAACGGAACGAAACC R:CCTGGCAATTGGAGATCAGT	5	0.51
MC07_92319	(**TC**)15	S13	GQ338440	(**TC**)15	F:GCAAATCAAAGAAGCCAAGC R:GTAGGGGTTGGGTTGATCCT	3	0.49
MC08_111602	(**CT**)11tctt(**TC**)6	S15	GQ338441	(**AG**)6(**AG**)12	–	–	–
MC01_5661	(**AG**)15	S18	GQ338442	(**AG**)15	–	–	–
MC01_3973	(**AG**)11	S20	GU166222	(**TC**)12	F:GCTCAAACTTTTGGGCTGAG R:AAGCTTGAGCTCCATTTCCA	2	0.20
MC11_158010	(**AG**)11	S24	GQ338443	(**CT**)11	F:GTCCAAAAATGGAGGCAAAA R:TTGTAGTGGAGGGGATCGAG	4	0.58
MC06_75265	(**GA**)13	S26	GQ338444	(**GA**)13	F:GCGGTAAATTCAGAATCCCA R:CCTGCTAGTTTGGCTTTTCG	5	0.58
MC06_73242	(**AG**)15	S32	GQ338447	(**TC**)17	F:AGCGAAGCAGCTTTATCGAG R:CGAAACGCACTATTCCCATT	16	0.70
MC11_160743	(**CT**)20	S33	GQ338448	(**GA**)19	F:CAGAGCTGGGTCTGTTGTGA R:AAATAATTTAGTGGGGCGGG	4	0.45
Mean						5.30	0.54


*A total of 27 SSR motifs that were assayed in two previous studies (Wang et al., 2010; Ji et al., 2012) were verified in the ‘Dali-11’ bitter gourd genome. The consistent or complementary motifs between our results and published reports are shown in bold font. ^∗^Indicates no or multiple blast hints.*

### Application of SSR Primers in Genetic Diversity Analysis

In this study, 21 primer pairs for markers that have motifs consistent with those of previous reports were used for the genetic diversity assessment of 211 bitter gourd (*M. charantia*) samples and one *M. balsamina* sample. All primer pairs successfully amplified multiple bands, ranging from 2 (MC01_3973) to 16 (MC06_73242) across the bitter gourd population. In total, 122 alleles were detected with a mean number of 5.30 alleles per locus (**Table 3**). PIC values of each marker ranged from 0.20 (MC01_3973) to 0.84 (MC08_106502), with a mean value of 0.54.

**FIGURE 4:**
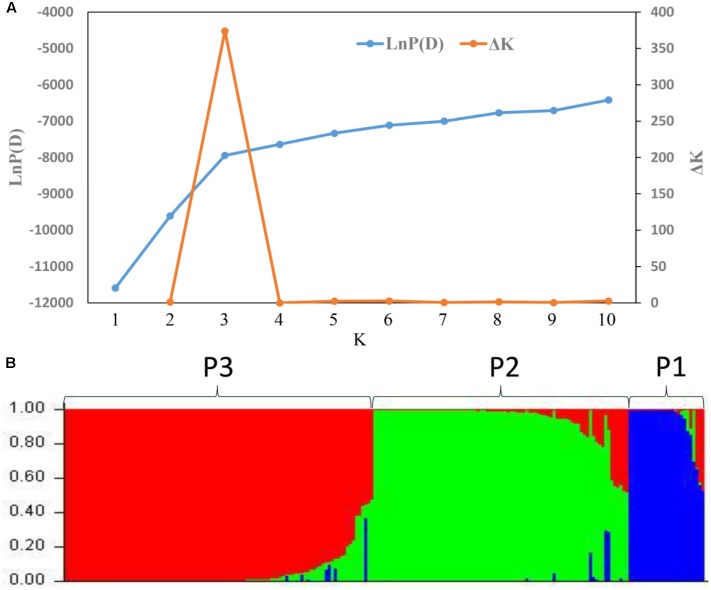
Analysis of the population structure based on 21 SSR markers. **(A)** Estimated LnP (*D*) and Δ*K* of a total of 211bitter gourd samples over 20 runs for each *K* value. **(B)** Classification of the 211 bitter gourd samples into three populations using STRUCTURE 2.3.4.

**FIGURE 5:**
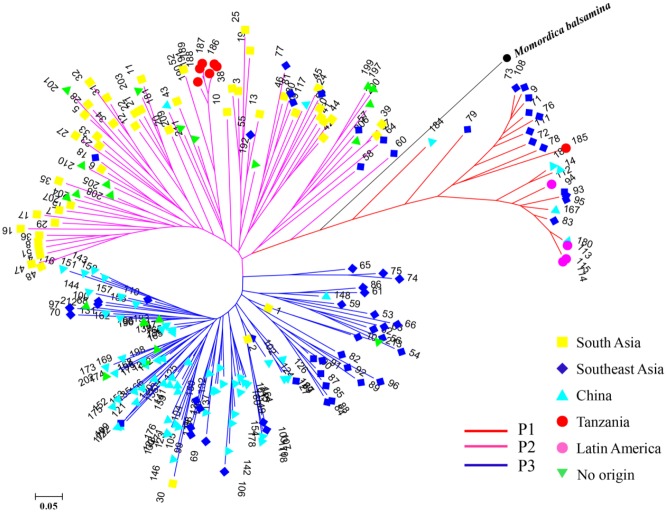
A NJ tree of 211 bitter gourd samples and one *Momordica balsamina* sample. “No origin” indicates samples were acquired from AVRDC and seed companies.

STRUCTURE was used to infer population structure of the bitter gourd samples (for *K* = 2, 3…, 10). At *K* = 3, the clustering of samples was the most appropriate because it produced the highest Δ*K* value (373.87; **Figure 4A**). Accordingly, the total panel was divided into three main populations that were labeled P1, P2, and P3, consisting of 23, 85, and 103 samples, respectively (**Figure 4B** and Supplementary Table S1). This result was consistent with the NJ tree and geographic origins of the samples (**Figure 5**).

From a geographic origin perspective, samples from Southeast Asia had wider distributions that were mainly localized in the P1 and P3 populations. Samples from South Asia and China were largely divided, and mainly grouped with P2 and P3, respectively. All four samples from Latin America were included in P1. One and six samples from Tanzania were assigned to P1and P2, respectively. Additionally, all the samples from AVRDC and seed companies were localized in P2 and P3 populations (**Figure 5**).

## Discussion

We examined the distribution of SSRs (composed of 1–6 bp) within seven genomes from four cucurbit species. Similar to other plant species (Cheng et al., 2016), the number of SSR motifs in cucurbits was positively correlated with genome size (*r* = 0.92, *P* < 0.01). Conversely, SSR density is usually negatively correlated with genome sizes (Deng et al., 2016); however, this trend was not significant within cucurbit genomes (*r* = –0.43, *P* = 0.34). Notably, cucurbit genomes are more compact, which is consistent with our results showing that the SSR density of four cucurbit species was comparable to that of *Arabidopsis* and rice, but two-fold higher than that of pepper, tomato, and potato (Cheng et al., 2016). Therefore, the abundance and high density of SSRs in cucurbits should make them an even more attractive type of molecular markers for genetic analysis in this family. Moreover, this comprehensive cucurbit SSR data set will enable the construction of an online repository for cucurbit SSR markers similar to the PlantFuncSSRs platform (Gaurav et al., 2016).

We first compared the classified types of SSR motifs among cucurbit genomes. Overall, bitter gourd had more motif types than did the other three cucurbits. Whether this difference is related to the complexity or evolution of the genomes of each species is unclear. Variation in selective constraint on sequence repeats might differ among the SSR motifs (Ellegren, 2004). In general, AT-rich motifs occur more frequently in dicotyledonous plants (Morgante et al., 2002; Cavagnaro et al., 2010; Cheng et al., 2016). We have observed similar results in (dicotyledonous) cucurbit genomes. Within each species, the classified motif types were not completely identical, which was demonstrated by comparisons among bitter gourd, cucumber, and watermelon genomes in the present work. A similar non-identity was also reported in two pepper genomes (Cheng et al., 2016). Compared to the ‘OHB3-1’ genome, the ‘Dali-11’ genome had more motif types, which may be partly explained by the greater genome assembly size of the latter one.

The genome-based SSR development is far more than the efficiency of traditional methods, including FIASCO (Wang et al., 2010; Guo et al., 2012) and SSR-enriched fragments or libraries (Ji et al., 2012; Saxena et al., 2015). Most SSRs developed by FIASCO (Wang et al., 2010) and from SSR-enriched fragments (Ji et al., 2012) showed motifs that were consistent with our results. Inconsistencies between our markers and those generated in previous studies may be owing to the differences among individual genomes. We synthesized 21 primers to amplify markers with consistent motifs for the purpose of genotyping 211 bitter gourd samples. Markers with these motifs were selected because they have displayed polymorphism in previous studies (Wang et al., 2010; Ji et al., 2012). In this study, most of these markers were also confirmed to have many alleles in the bitter gourd population and were demonstrated to be highly informative (mean PIC = 0.54) according to a standard threshold for discriminating power (Botstein et al., 1980).

Because of limited germplasm collection and molecular marker availability, scant attention has been paid to studying the genetic diversity of bitter gourd. Until recently, the best characterization of genetic diversity in bitter gourd was conducted using 114 bitter gourd accessions that originated from South and Southeast Asia by using 50 SSR markers (Dhillon et al., 2016). We collected a set of 211 samples originating from a broader array of geographic regions, including a unique group from China in particular. Both studies divided the germplasm materials into three main populations. This broad sampling enabled us to demonstrate that bitter gourds from South Asia and China represent two relatively distinct genetic reservoirs of molecular variation, providing new insight into the relationship between molecular diversity and geographic distributions in an important crop. This assessment of diversity indicated that the SSR markers developed in this study will be a valuable marker resource for future bitter gourd research.

## Conclusion

In this study, we identified 129,947 to 331,062 SSR motifs among four cucurbit genomes, including 188,091 and 167,160 SSR motifs within the bitter gourd ‘Dali-11’ and ‘OHB3-1’ genome, respectively. Of these, the dominant SSR motif type in cucurbits is rich in A/T, AT/AT, AAT/ATT, and AAAT/ATTT. A large set of 138,727 *in silico* unique SSR primer pairs were designed for bitter gourd based on ‘Dali-11’ reference genome. A selected set of 71 SSR primer pairs were validated by PCR amplification with a successful rate of 100.00%. We then used 21 polymorphic SSR markers to assess the genetic diversity of 211 bitter gourd samples, yielding three distinct populations that displayed clear geographic differentiation. These genome-wide SSR markers and the clustering results for the bitter gourd panel will play an important role in bitter gourd genetic improvement and breeding programs.

## Author Contributions

JuC, JiC, and KH conceived and designed the experiments. DN, QZ, and ND contributed to germplasm collection. JuC and JP performed the experiments. YH and WH performed the bioinformatics analyses. JuC and JiC wrote the manuscript, and HK revised the manuscript. All authors read and approved the final manuscript.

## Conflict of Interest Statement

The authors declare that the research was conducted in the absence of any commercial or financial relationships that could be construed as a potential conflict of interest.

## Footnotes

1 http://pgrc.ipk-gatersleben.de/misa/

2 http://pgrc.ipk-gatersleben.de/misa/primer3.html

## Supplementary Material

**FIGURE S1 |** The validation of amplification using 50 newly developed SSR markers. **(A)** marker numbers 1 to 11; **(B)** marker numbers 12 to 23; **(C)** marker numbers 24 to 35; **(D)** marker numbers 36 to 47; **(E)** marker numbers 48 to 50. The primer sequences are listed in Supplementary Table S7. Two bitter gourd samples were genotyped in this validation. (a) bitter gourd sample ‘Dali-11;’ (b) bitter gourd sample ‘K44.’

**FIGURE S2 |** Amplification of 21 SSR markers used in population genetic analysis. **(A)** marker numbers 1 to 9; **(B)** marker numbers 10 to 21. The primer sequences are listed in **Table 3**. (a) bitter gourd sample ‘Dali-11;’ (b) bitter gourd sample ‘K44.’
