# Supplementary figures and images for: Genome-Wide Analysis of Simple Sequence Repeats in Bitter Gourd (Momordica charantia)
Source: Front Plant Sci. 2017 Jun 22;8:1103. doi: 10.3389/fpls.2017.01103 (PMC5479929; doi:10.3389/fpls.2017.01103)

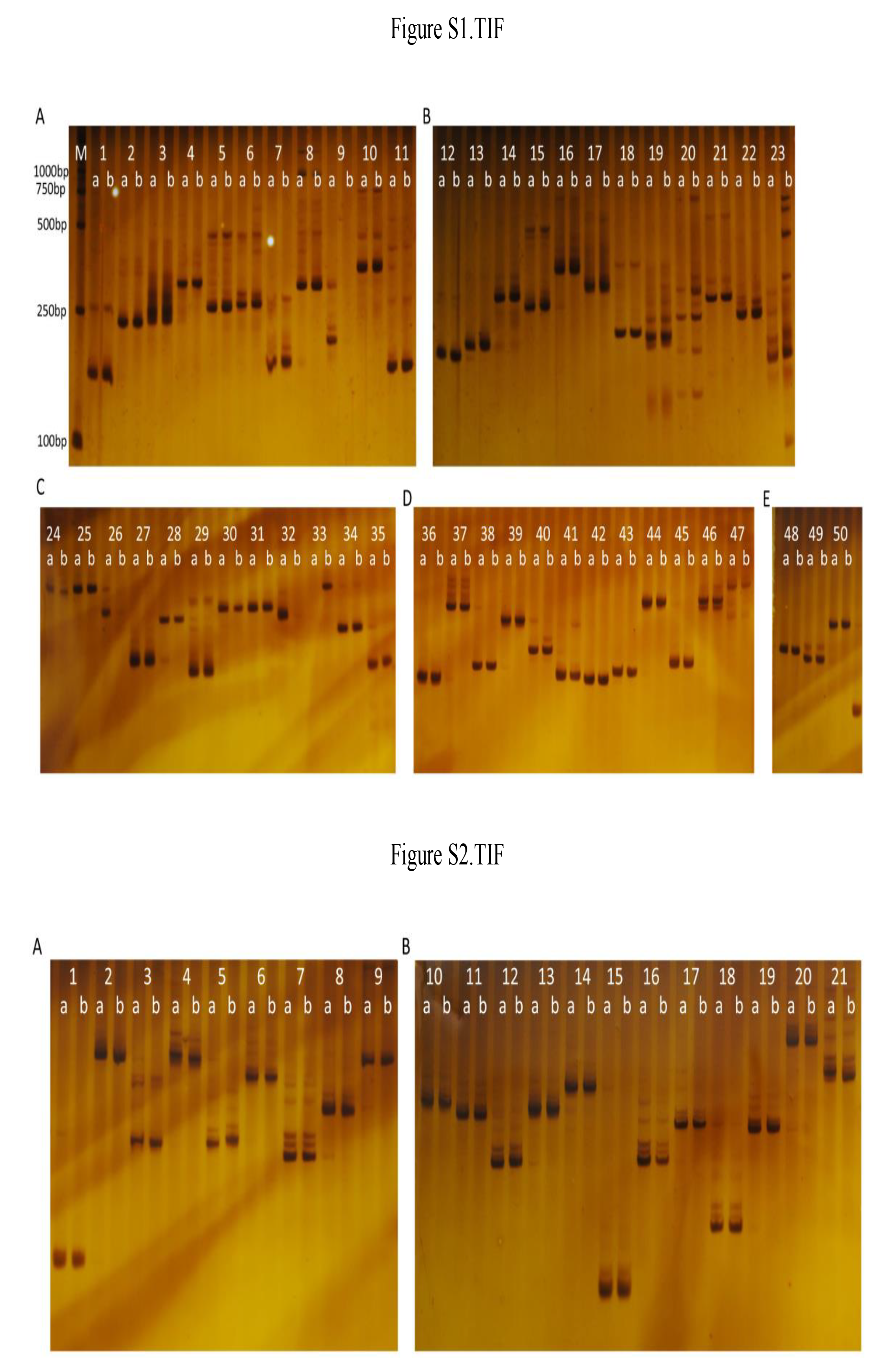

Supplement: Supplementary file 1 [file Images_1_and_2.TIF]
