# Supplementary material for: Genome-Wide Analysis of Simple Sequence Repeats in Bitter Gourd (Momordica charantia)
Source: Front Plant Sci. 2017 Jun 22;8:1103. doi: 10.3389/fpls.2017.01103 (PMC5479929; doi:10.3389/fpls.2017.01103)
Supplement: Supplementary file 3 [file Tables_2_and_3.DOCX]

**Supplementary Table S2. List of seven different genomes for SSR identification in the present study.**

| Name of variety | Species | Chromosome number | Total size of examined sequences (bp) | Web sources |
| --- | --- | --- | --- | --- |
| Dali-11 | *M. charantia* | 11 | 294,014,906 | Unpublished |
| OHB3-1 | *M. charantia* | 11 | 285,613,949 | <https://www.ncbi.nlm.nih.gov/Traces/wgs/?val=BDCS01#contigs> |
| 9930 | *C. sativus* | 7 | 197,271,687 | <ftp://www.icugi.org/pub/genome/cucumber/Chinese_long/v2/> |
| PI183967 | *C. sativus* var. *hardwickii* | 7 | 204,803,225 | <ftp://www.icugi.org/pub/genome/cucumber/PI183967/> |
| 97103 | *C. lanatus* | 11 | 355,247,419 | <ftp://www.icugi.org/pub/genome/watermelon/97103/v1/> |
| WCG | *C. lanatus* | 11 | 404,684,803 | <ftp://www.icugi.org/pub/genome/watermelon/WCG/v1/> |
| DHL92 | *C. melo* | 12 | 406,930,216 | <https://melonomics.net/files/Genome/Melon_genome_v3.5.1/> |
| Total |  |  | 2,148,566,205 |  |

**Supplementary Table** S**3. Primer pairs for 50 new SSR markers developed from unassembled scaffolds.**

| Number | SSR ID | Forward primer(5'-3') | Reverse primer(5'-3') |
| --- | --- | --- | --- |
| 1 | scaffold31_168189_1 | CGATTGCTTAGGGCATGTTT | TAATGCAAACCCGTCAATCA |
| 2 | scaffold58_168793_1 | CTGGAGCAACAGCAAAGACA | GGGCGTTACACGTTGAACTAA |
| 3 | scaffold60_169440_2 | AACCGACATCAAACCGACTC | GCAGGCTCCTGTGAAATAGC |
| 4 | scaffold10_170000_1 | TCCTGCATAGGTTTGGGAAC | GCGCCAAATGCATAGTTCTT |
| 5 | scaffold16_170517_1 | GTTGAAGTTATGAAAAGTGGCTGAC | AATTCCGGTTCAAACAATGC |
| 6 | scaffold15_170930_1 | AGATCGATTCTTGGCTTCCT | GTTGCTGTGAAAGAGCACCA |
| 7 | scaffold18_171500_1 | ATTCCACTCCCCCAAACTTC | TGGGTACACTTTGGATTGTGAG |
| 8 | scaffold20_172030_1 | AAGAACCGGCCAATTTTCTT | GAAAATTGCACAAACCACCC |
| 9 | scaffold99_172450_2 | GGATACCCAGAAAAACGCAA | CCCTGACGTGGGGTAACTAA |
| 10 | scaffold25_172780_1 | CAATTTCATCCCTATGGTTTGG | GGAGCTTTCGTAGAGCATGG |
| 11 | scaffold86_173209_4 | CTTCTTCGAGCGTTTTTCTTTC | CAATTTCCTAGTGGCCAACAT |
| 12 | scaffold94_173680_1 | AAGGGGGAGGGGAAAATACT | CCTTTTATGAGTGGGTTTCCA |
| 13 | scaffold17_173804_1 | TTCCAAGCATCCTCAACACA | AAGGCACCGACAAGAAAATG |
| 14 | scaffold113_174210_1 | CCCACGAAATAAACGTCACC | GCCCTGAACGAGATTATGGA |
| 15 | scaffold109_174500_1 | TGGTGCCAAACACCATAAAA | TGAGTACGAGAATCCCCCAC |
| 16 | scaffold7_175010_1 | TTGCATGGTTTCATGTCGTT | ACACGTGTCCTATTCGGGAG |
| 17 | scaffold76_175380_1 | CGAGCTTGGTTCATCTTGGT | TTGACCACGGTAAAATTAGTGG |
| 18 | scaffold163_175681_1 | GCCGAGAGGAGAGGAAGAAT | TTTGGGCTTTGTGCATGATA |
| 19 | scaffold107_176000_1 | GTAACCAATCCGAGCCTGTC | AGGGAGTGGCTCCTGAATTT |
| 20 | scaffold59_176370_4 | TATGACTCGAACCAACACGC | GCGGTGATAATGACGGAGTT |
| 21 | scaffold90_176690_1 | AAGAAGTGAAATCGGGGGTT | AGCTGCTGTGGTGATTGTTG |
| 22 | scaffold138_176849_1 | GGTGTTTCGATGTCCCAGTT | AAGGTGTGTGCATCAAGCTG |
| 23 | scaffold196_177411_1 | TTCCCACCTTAGTGGACAGC | TCGCTAGGTTTACAAATCCACA |
| 24 | scaffold157_178088_4 | TGAAACAATACAATGGCCGA | CTTAGCCCTGTTTATGGGCA |
| 25 | scaffold127_178420_1 | ACCTGTGAAAGGCAGAGGAA | CACGCTCCAACCTAGAATGTG |
| 26 | scaffold19_178554_1 | TCGAGTAGCTGATTGAGCCC | TTATTTGCACTTTCTGGCCC |
| 27 | scaffold144_178708_5 | GGTGGCTGGTTTATCGAAGA | AGTTGGGCCTCATTTAAGGG |
| 28 | scaffold102_178904_1 | GTCCCCATTCCCATTTCTTT | CAATGTTCCGCATTTCACTG |
| 29 | scaffold124_179069_1 | AGCTCCATACCGAGGAGGAT | AAAAATGTTGGGAGAAAGGC |
| 30 | scaffold180_179315_2 | TTCCAGACCTTCAATTTCCG | CTTGACAATGCGAGTCAAAGTT |
| 31 | scaffold8_179478_1 | CATTTGGGTTCGACATCTCC | TTCTTTCACGGGAGCTCAAC |
| 32 | scaffold162_179635_1 | ATGTCCTTTGTAACGCCTCG | AAGCCCAATCAACTTTCCCT |
| 33 | scaffold35_179753_2 | CGGTGGCGTTAGCATTAGAT | GAACCTCGTGCCACTTCATT |
| 34 | scaffold67_179835_1 | GTTGGGGTTATGGGTGAGTG | CCCACTACGTGCCAATTTCT |
| 35 | scaffold116_179973_1 | ACTGCTTCTGGGGGAACTTT | AGAGAGAGAGACGGCGACAG |
| 36 | scaffold52_180161_2 | GCTCCAGCATTTCCATAACC | ACCATGAGAGCCTTTCCCTT |
| 37 | scaffold119_180366_1 | TGGTTGTGTAACGCCTCGTA | GTAGGCGGCTGAAAACAAAA |
| 38 | scaffold146_180451_1 | TACCTGTGCGTCAAACTTGC | GAGGGTGACCTGTGGAAGAA |
| 39 | scaffold33_180559_1 | CAGTTTGGTCATTTTTAAGGGG | CAACCGGATCCTTCATGTTT |
| 40 | scaffold167_180760_1 | TTGGATGGTGGTTTGTGTGT | AGGCAAAAGGGGATTGATTT |
| 41 | scaffold104_180893_1 | AAGTGTGGAGCCTTCCATTG | ACCGGTTTTTGGCTTCCTAA |
| 42 | scaffold191_180996_1 | GTCCACATGGTCAGGCTCTT | ACAAGTCACGCTGTGCAACT |
| 43 | scaffold165_181201_1 | TGCTTTCACTCAACCAATGA | GCATTCACATCACTCGCATC |
| 44 | scaffold32_181368_1 | CCACGTTTTCCTCTCGTGTT | GGCCATTTTTGTGCTTTGTT |
| 45 | scaffold50_181474_1 | TCCCCTCCGATTTTCTCTTT | CAAACACGCTAAAGAATGCG |
| 46 | scaffold91_181520_2 | TGGGCATGTTTGACCATAAA | GACCCTCGTGTCCTCCATAA |
| 47 | scaffold230_181675_1 | GGTCACCATCTGCCTCACTT | TTCCCTGATGTCTCCCTCAC |
| 48 | scaffold280_181787_1 | TGGTTCTCTTTCCTGAAAACCT | AAATGTCCCTGGCTAGCAACT |
| 49 | scaffold145_182138_1 | AAGGAGAAAGCTTTAGCGCC | TCTTCCGTCTCTCCGTCCTA |
| 50 | scaffold126_182221_1 | CAGGTGAACAGAGAGGCACA | CGTGGATCAATTTTGTGTGA |
